# Microsurgical scleral drainage and trabeculectomy-scleral flap adjustable suture combination technique in the treatment of primary glaucoma

**DOI:** 10.12669/pjms.36.2.1439

**Published:** 2020

**Authors:** Li Xiahou, Chunlan Liu, Weihong Zhou, Shasha Yang

**Affiliations:** 1 Li Xiahou Department of Ophthalmology, Xinyu People’s Hospital, Xinyu, Jiangxi Province, 338000, China; 2 Chunlan Liu Department of Ophthalmology, Xinyu People’s Hospital, Xinyu, Jiangxi Province, 338000, China; 3 Weihong Zhou Department of Ophthalmology, Xinyu People’s Hospital, Xinyu, Jiangxi Province, 338000, China; 4 Shasha Yang The Second Affiliated Hospital, South China University of Technology, Guangzhou First People’s Hospital, Guangzhou, 510180, China

**Keywords:** Primary glaucoma, Trabeculectomy, Scleral flap adjustable suture, Scleral space drainage pool

## Abstract

**Objective:**

To investigate the clinical effect of microsurgical scleral drainage and trabeculectomy combined with scleral flap adjustable suture technique in the treatment of primary glaucoma.

**Methods:**

One hundred primary glaucoma patients (120 eyes) in Xinyu People’s Hospital of Jiangxi province were selected from July 2014 to June 2016. The patients were randomly divided into control group and study group. The control group was treated with compound trabeculectomy, while the study group was treated with microsurgical scleral drainage and trabeculectomy combined with scleral flap adjustable suture technique. In both groups of patients, intraocular pressure, functional filtering bleb formation, and complications before and after surgery were monitored for three days, one week, one month, three months, six months and one year, while anterior chamber depth was determined one week after operation. The extent of success of operation was compared between the two groups.

**Results:**

At three days, one week, one month, three months, six months and one year after surgery, intraocular pressure of study group was significantly lower than that of the control group (P<0.05). There was 93.33% formation of functional filtering blebs in the study group, which was significantly higher than that in the control group (60.00%, P<0.001). Moreover, normal anterior chamber formation was significantly higher in the study group (91.67%) than in the control group (71.67%, P<0.01). There was 95.00% operation success in the study group, relative to 68.33% success in the control group (P<0.001).

**Conclusion:**

Microsurgical scleral drainage and trabeculectomy combined with scleral flap adjustable suture technique has better curative effect on primary glaucoma than compound trabeculectomy. Moreover, it does not exacerbate complications. Therefore, the combination treatment technique merits clinical application.

## INTRODUCTION

Glaucoma is an eye disease with clinical characteristics of optic nerve degeneration and visual field defect, which are not readily detected, but are progressive and irreversible; 30% of people with glaucoma become blinded.^1^,^2^ Visual function in glaucoma patients is severely damaged, usually manifesting as optic atrophy and visual field defect which are serious threats to the visual function and daily lives of patients.^3^ Available clinical data show that the incidence of glaucoma in China is 20%-40%.^4^ Therefore, the development of newer and more effective treatment strategies for glaucoma is of immense clinical significance. Surgical treatment is the main method of treating primary glaucoma. Surgical treatment effectively reduces intraocular pressure and protects and saves visual function.^5^ Trabeculectomy, the standard therapy for glaucoma, has a beneficial effect of lowering intraocular pressure.^6^,^7^ However, clinical practice has revealed that in the course of treatment, patients’ filters are susceptible to tissue edema,^8^ fibroplasia and wound repair, which may result in blockage of filters, interruption of drainage, and ultimately operation failure. Moreover, the incidence of operation-related complications may be high, resulting in poor therapeutic outcomes. Trabeculectomy combined with scleral flap adjustable suture technique is an improvement on the trabeculectomy technique.^9^ The application of scleral flap adjustable suture technique forms anterior chamber early, to reduce the incidence of shallow anterior chamber and choroidal detachment. Moreover, it achieves quantitative adjustment of aqueous humor filtration volume by controlling the time of suture removal and number of removed sutures, thereby forming ideal functional filtration blebs, leading to ideal intraocular pressure control, although connective tissue proliferation can still lead to surgery failure.^10^ In recent years, it has been reported that trabeculectomy combined with sclera pool not only establishes a lasting outflow channel of aqueous humor and solves the problem of early excessive filtering after surgery, but also prevents scleral flaps from adhering to the scleral bed, thereby effectively preventing scar formation.^11^

The purpose of this study was to investigate the clinical effect of microsurgical scleral drainage and trabeculectomy combined with scleral flap adjustable suture technique in the treatment of primary glaucoma, with a view to providing reference for clinical practice.

## METHODS

One hundred patients (120 eyes) with primary glaucoma who were hospitalized in Xinyu People’s Hospital of Jiangxi province from July 2014 to June 2016 were selected. All patients met the diagnostic criteria of primary glaucoma.^12^ They were divided into control group and study group, using the random number table. There were 52 cases (60 eyes) in the study group, and 48 cases (60 eyes) in the control group. In all, there were 62 cases of acute primary angle closure glaucoma (APACG) (68 eyes), 28 cases of chronic primary angle closure glaucoma (CPACG) (36 eyes), and 10 cases of primary open angle glaucoma (POAG) (16 eyes). Patients in the study group were treated with microsurgical scleral drainage and trabeculectomy combined with scleral flap adjustable suture technique. In contrast, compound trabeculectomy was performed in the control group. The angle of anterior chamber was re-examined before operation. The dynamic angle of the chamber was still lower than 180 degrees after lowering of intraocular pressure and pupil contraction. There were no significant differences in sex, age, baseline intraocular pressure and type of disease between the two groups (P>0.05, Table-I).

**Table-I:** General data of the study group and the control group.

Group		Study group	Control group	X^2^/t value	P value
Patients (n)		52	48	/	/
Eyes (n)		60	60	/	/
Male/Female (n/n)		21/31	20/28	0.017	>0.05
Glaucoma type	APACG	35	33	0.003	>0.05
	CPACG	17	19		
	POAG	8	8	
Ages		58.13±13.23 (45-79)	57.59±11.82 (43-81)	0.235	>0.05
Intraocular pressure(mmHg)		50.51±5.91	49.82±6.25	0.621	>0.05

### Inclusion criteria:

Diagnosis of APACG, CPACG and POAG consistent laid down creteria,^13^ and provision of informed consent by the patient for operation after being duly informed about relevant treatments and surgical scheme.

### Exclusion criteria:

Patients who previously underwent trabecular surgery twice, and patients who underwent combined surgery were excluded. Moreover, patients with refractory glaucoma, secondary glaucoma, developmental glaucoma, severe cardiovascular diseases or other severe organic diseases, severe diseases in the immune system, as well as those with a history of psychiatric or nervous system disease, were also excluded. This study was reviewed and approved by the medical ethical committee (Dated December 11, 2018) of the hospital.

### Surgical methods:

All operations in the control group and study group were carried out by the same surgical team with bedside clinicians as surgical assistants. The operation procedures in the study group were as follows: After conventional disinfection and spreading of aseptic towel, surface and subconjunctival anesthesia were performed. The eyelid was opened using an eye speculum. The superior rectus was fixed. High-position conjunctival flap which took corneal limbus as the fundus, was made at the site which was 8-10 mm above corneal limbus in the direction of 12 o’clock position. The Tenon’s capsule was separated to expose an 8×7 mm sclera surgical field. The blood vessels on the surface of the sclera were burned to stop bleeding. Then, a 5 mm×6 mm scleral flap which took the corneal limbus as the fundus, and was one-third the thickness of the sclera, was made. A sclera tunnel knife was used to separate forward to the site which was one mm inside transparent cornea. Then, a 3×3 mm middle layer scleral flap of thickness one-third that of the sclera was removed, taking the corneal limbus as the fundus in the middle below the shallow scleral flap.

A 4 mm×3.5 mm cotton fleexe immersed in 0.25-0.33 mg/mL mitomycin C was placed below the scleral flap and conjunctival flap for 2-3 minutes. Then, the surgical field and cornea were washed with 150 mL of BBS. An anterior chamber puncture was made on the corneal limbus at the 9 o’clock position using a paracentesis knife. Aqueous fluid was slowly released via the puncture mouth so as to further reduce the intraocular pressure. Then, 1.5×1.5 mm trabecular meshwork and deep corneosclera tissue below the scleral flap were removed along the posterior margin of sclera ridge. This was followed with peripheral iridectomy. The top of each side of the scleral flap was sutured (one stitch) with 10/0 non-absorbable nylon thread. A small amount of BBS was injected via the puncture mouth of the anterior chamber to reconstruct the anterior chamber, and some aqueous fluid was discharged below the scleral flap. Then, two adjustable sutures were made at the bottom of the scleral flap. The degree of tightness was adjusted, and the tightness was suitable when the seepage below the scleral flap slowly emerged to maintain the depth of the anterior chamber, or when the intraocular pressure was normal or low. Tenon’s capsule was sutured (2-3 stitches) using 10-0 non-absorbable nylon thread. The incision on the bulbar conjunctiva was continuously sutured using 10-0 non-absorbable nylon thread. Dexamethasone (5 mg) was injected subconjunctivally, and erythromycin eye ointment was used. The operated eye was bound up. After surgery (usually 3-14 days), the two adjustable sutures were removed, depending on the value of intraocular pressure and chamber pressure.

Compound trabeculectomy was used in the control group. The operation was performed under a microscope. Surface and subconjunctival anesthesia were performed. The eyelid was opened using an eye speculum. The superior rectus was fixed. The Tenon’s capsule was separated to expose a 8×7 mm sclera surgical field. Then, a 4 mm×5 mm scleral flap which took the corneal limbus as the fundus, and was one-third of sclera thickness, was made. A sclera tunnel knife was used to separate forward to the site which was one mm inside transparent cornea. A 4 mm×3.5 mm cotton fleexe immersed in 0.25-0.33 mg/mL mitomycin C was placed below the scleral flap and conjunctival flap for 2-3 minutes. Then, the surgical field and cornea were washed with 150 mL of BBS. An anterior chamber puncture was performed using a paracentesis knife to slowly release aqueous fluid to achieve slow decrease of intraocular pressure. Trabecular meshwork and deep corneosclera tissue below the scleral flap were removed along the posterior margin of sclera ridge. Then, peripheral iridectomy was performed. The top and bottom on both sides of the scleral flap were sutured (one stitch). The conjunctival incision was continuously sutured, and 5 mg of dexamethasone was subconjunctivally injected. Erythromycin eye ointment was applied, and the operative eye was bound up.

### Clinical indicators

The indices determined were intraocular pressure and formation of functional filter-bubble before surgery, and three days, one week, one month, three months, six months and one year after surgery. Moreover, the depth of anterior chamber at one week after operation was determined.

### Statistical method

Statistical analysis was performed using SPSS 17.0. Measurement data are expressed as mean±standard deviation. Two-group comparisons were done using t-test. Enumeration data are expressed as percentage. Statistical differences in enumeration data were compared between the two groups using Chi-square test. Differences were considered statistically significant at P<0.05.

## RESULTS

There was no significant difference in the preoperative intraocular pressure value between the study group and control group (P>0.05). However, the intraocular pressure of the study group was significantly lower than that of the control group (P<0.05, Table-II).

**Table-II:** Intraocular pressure at different time periods before and after the operation in the study group and the control group (mmHg).

Group	Study group	Control group	t value	P value
Before	50.51±5.91	49.82±6.25	0.621	>0.05
After three days	11.41±2.37	13.15±2.82	3.659	<0.05
After one week	12.86±2.05	14.30±2.87	2.943	<0.05
After one month	12.42±2.18	15.61±3.54	5.944	<0.05
After three months	12.18±2.53	17.44±4.46	7.946	<0.05
After six months	12.36±2.64	20.73±5.81	10.159	<0.05
After twelve months	13.47±4.12	21.88±6.26	8.962	<0.05

The extent of formation of functional filtering blebs in the study group was significantly higher than that in the control group (P<0.001). Table-III,

**Table-III:** Comparison of filtering blebs at one year after operation.

Group	Functional filtering blebs		Non-functional filtering blebs	
	I-type	II-type	III-type	VI-type
Study group	11(18.33%)	45(75.00%)	3(5.00%)	1(1.67%)
Control group	9(15.00%)	27(45.00%)	17(28.33%)	7(11.67%)
X^2^ value	18.634			
P value	<0.001			

One week after the operation, the extent of formation of normal anterior chamber in the study group was significantly higher than that in the control group (P<0.01, Table-IV). There were no significant differences in the incidence of postoperative complications between the two groups (P>0.05, Table-V).

**Table-IV:** Comparison of the onset of anterior chamber in the study group and the control group one week after the operation.

Group	Study group	Control group	X^2^ value	P value
Normal	55(91.67%)	43(71.67%)	8.015	0.005
I-grade	5(8.33%)	14(23.33%)		
II-grade	0(0%)	3(5.00%)		
III-grade	0(0%)	0(0%)		

**Table-V:** Comparison of incidence of complications between the study group and control group.

Group	Study group	Control group	X^2^ value	P value
Postoperative corneal edema	7(11.67%)	6(10.00%)	1.174	0.965
Fibrinous exudate in pupillary area and anterior chamber inflammation	3(5.00%)	2(3.33%)		
Postoperative low intraocular pressure	2(3.33%)	3(5.00%)		
Filtering bleb leak	1(1.67%)	0(0%)		
Malignant glaucoma	0(0%)	2(3.33%)	/	/

In the study group, operation was successful in 57 eyes (95.00%) comprising 54 eyes which were completely successful (90.00%) and 3 eyes which were conditionally successful (5.00%). Three eyes failed (5.00%). In the control group, operation was successful in 41 eyes (68.33%), comprising 34 eyes which were completely successful (56.67%) and 7 eyes which were conditionally successful (11.67%); 19 eyes failed (31.67%). The success of operation in the study group was significantly higher than that in the control group (X^2^=14.249, P<0.001).

## DISCUSSION

Primary glaucoma is divided into PACG and POAG, based on differences in pathogenesis and anatomical structure of angle of chamber. Moreover, depending on degree of onset and clinical course, PACG is sub-divided into APACG and CPACG.^14^-^16^ It has been reported that PACG is the most common form of glaucoma in China.^17^ The control of intraocular pressure is the only effective method of treating glaucoma at present.^18^ Filtering surgery is one of the methods for glaucoma treatment. This entails the creation of an artificial drainage for aqueous humor through surgery so as to reduce intraocular pressure.

Trabeculectomy is the traditional and the most common and classical surgical method for glaucoma treatment. Through this operation, aqueous humor can be drained to the subconjunctival space and absorbed to form follicles.^19^ The high degree of failure in trabeculectomy (20%-30%), limits its application.^20^ Fibrous hyperplasia, tissue edema and wound repair are the causes of failure of trabeculectomy. When the filters are blocked, drainage is interrupted. Then, the filters become adhesive, leading to difficultly in formation of functional follicles.^21^ A clinical study found that mitomycin C adjuvant therapy with trabeculectomy had a significant effect. Mitomycin C is an antineoplastic antibiotic derived from Streptomyces cephala.^22^ It significantly inhibits the proliferation of fibroblasts and reduces the formation of scars. Its pharmacological effect is significant. Mitomycin C adjuvant therapy effectively alleviates the scarring of filter passage after operation, although it can also result in the occurrence of shallow anterior chamber post-surgery. Shallow anterior chamber causes serious consequences such as bullous keratopathy, iris adhesion, malignant glaucoma, cataracts, choroidal hemorrhage and detachment due to damaged corneal endothelial cells, leading to failure of operation and abnormal intraocular tissue structure. The use of adjustable suture for scleral flaps solves the problem of shallow anterior chamber caused by over filtration in the early stage, resulting in significant, reduction of post-surgery complications and improvement in degree of operation success. However, in the long run, intraocular pressure may increase again due to the proliferation of connective tissue at the scleral opening at the angle of the chamber, and the inhibition of aqueous humor outflow induced by the healing of scleral lamellar wound. These can lead to failure of operation. In recent years, it has been found that trabeculectomy combined with scleral pool effectively establishes a lasting outflow channel for aqueous humor, reduces intraocular pressure, prevents scleral flap from adhering to scleral bed, and reduces scarring.^23^ Therefore, trabeculectomy was combined with adjustable sutured flap, in order to artificially control the depth of anterior chamber and intraocular pressure after trabeculectomy, and improve the success of surgery.

The results of this study showed that trabeculectomy in combination with adjustable sutured flap effectively reduced intraocular pressure in short term and long term, and promoted the formation of functional filtering blebs, when compared with compound trabeculectomy. It might be due to enhanced discharge of aqueous humor from the conjunctiva, and also through uveosceleral outflow in the short-term study group. Moreover, the intraocular pressure was maintained at a normal level for a long time in the study group because of the formation of filtration channel scar. The incidence of shallow anterior chamber in the study group was low, probably because adjustable sutures artificially control the filtration volume, and adjusting the suture tightness controlled the outflow resistance of aqueous humor after passing through the dangerous shallow anterior chamber. Results of the follow-up showed that operation success in the study group was significantly higher than that in the control group, which was in line with expectations.

## CONCLUSION

The application of microsurgical scleral drainage and trabeculectomy in combination with scleral flap adjustable suture technique in primary glaucoma effectively controls early filtration flow and speed of aqueous humor, and reduces the incidence of low intraocular pressure and shallow anterior chamber caused by strong filtration. Moreover, it reduces scars, promotes the formation of functional filtering blebs and the establishment of permanent aqueous humor filtration channels, and improves the success of glaucoma surgery. Therefore, this combination technique merits clinical application and promotion.

### Authors’ Contribution:

**LXH & SSY:** Study design, data collection and analysis.

**CLL, WHZ & SSY:** Manuscript preparation, drafting and revising.

**LXH & SSY:** Review, final approval of manuscript, are responsible for integrity of research.
